# Members of the miRNA-200 Family Regulate Olfactory Neurogenesis

**DOI:** 10.1016/j.neuron.2007.11.018

**Published:** 2008-01-10

**Authors:** Philip S. Choi, Lisa Zakhary, Wen-Yee Choi, Sophie Caron, Ezequiel Alvarez-Saavedra, Eric A. Miska, Mike McManus, Brian Harfe, Antonio J. Giraldez, Robert H. Horvitz, Alexander F. Schier, Catherine Dulac

**Affiliations:** 1 Howard Hughes Medical Institute, Department of Molecular and Cellular Biology, Harvard University, Cambridge, MA 02138, USA; 2 Department of Molecular and Cellular Biology, Harvard University, Cambridge, MA 02138, USA; 3 Howard Hughes Medical Institute, Department of Biology, Massachusetts Institute of Technology, Cambridge, MA 02139, USA; 4 Diabetes Center, University of California, San Francisco, San Francisco, CA 94143, USA; 5 Department of Molecular Genetics and Microbiology, University of Florida, Gainesville, FL 32611, USA; 6 Division of Sleep Medicine, Center for Brain Science, Harvard Stem Cell Institute, Broad Institute, Cambridge, MA 02139, USA; 7 Genetics Department, Yale University School of Medicine, New Haven, CT 06520, USA

**Keywords:** DEVBIO, MOLNEURO

## Abstract

MicroRNAs (miRNAs) are highly expressed in vertebrate neural tissues, but the contribution of specific miRNAs to the development and function of different neuronal populations is still largely unknown. We report that miRNAs are required for terminal differentiation of olfactory precursors in both mouse and zebrafish but are dispensable for proper function of mature olfactory neurons. The repertoire of miRNAs expressed in olfactory tissues contains over 100 distinct miRNAs. A subset, including the miR-200 family, shows high olfactory enrichment and expression patterns consistent with a role during olfactory neurogenesis. Loss of function of the miR-200 family phenocopies the terminal differentiation defect observed in absence of all miRNA activity in olfactory progenitors. Our data support the notion that vertebrate tissue differentiation is controlled by conserved subsets of organ-specific miRNAs in both mouse and zebrafish and provide insights into control mechanisms underlying olfactory differentiation in vertebrates.

## Introduction

MicroRNAs (miRNAs) constitute a large class of small noncoding RNAs that provide multicellular organisms with elaborate yet poorly understood strategies for posttranscriptional gene regulation (Bartel, 2004). Hybridization to fully or partially complementary sequences enables miRNAs to specifically direct degradation or translational inhibition of target transcripts (Plasterk, 2006). Genetic analyses in invertebrate systems have identified essential roles for miRNAs in the regulation of various developmental processes, including specific steps of neuronal differentiation. In *C. elegans*, *lsy-6* and *miR-273* have been reported to participate in negative-feedback loops that ensure asymmetric expression of taste receptors in chemosensory neurons (Chang et al., 2004; Johnston and Hobert, 2003). In *Drosophila*, miR-7 has been implicated in photoreceptor cell differentiation through regulation of local EGF receptor signaling (Li and Carthew, 2005). The essential roles played by some miRNAs in controlling invertebrate neurogenesis and the dynamic patterns of miRNA expression during vertebrate development have raised the issue as to whether miRNAs might similarly regulate aspects of vertebrate neural development (Miska et al., 2004; Kosik and Krichevsky, 2006; Cao et al., 2006; Makeyev et al., 2007). This question has remained unanswered because loss-of-function studies of specific neural microRNAs in vertebrates have not yet been performed.

Thanks to its molecular and genetic tractability, the process of olfactory neurogenesis offers a unique opportunity to uncover regulatory networks underlying neuronal specification and differentiation. The main olfactory epithelium (MOE) of mammals is a pseudostratified epithelium, which extends from an underlying basal lamina to the lumen of the nasal cavity. Olfactory neurogenesis in rodents is initiated at midgestation with the thickening and invagination of the bilaterally symmetric olfactory placodes. The posterodorsal recess of the placodal epithelium differentiates into a mature, self-regenerating sensory epithelium that contains a highly heterogeneous and constantly renewing population of neurons and neuronal precursors (reviewed in Dulac and Zakhary, 2004). Adult MOE contains three major cell groups: basal cells, olfactory sensory neurons (OSN), and supporting cells. The basal cells are a population of dividing cells located adjacent to the basal lamina that continuously generate olfactory progenitors, which in turn differentiate into olfactory neurons. In the mouse, each mature olfactory sensory neuron expresses a unique olfactory receptor gene from a large family of approximately 1000 genes such that all neurons expressing the same receptor transcript are randomly dispersed within one of four broad zones of the olfactory epithelium (Buck and Axel, 1991; Chess et al., 1994; Malnic et al., 1999; Ressler et al., 1993; Vassar et al., 1993).

What are the transcriptional regulators underlying such sensory diversity? Genetic analysis of the olfactory epithelium has pointed to the essential role played by basic helix-loop-helix (bHLH)-containing transcription factors related to the *Drosophila* proteins achaete-scute and atonal in controlling MOE development (reviewed in Bertrand et al., 2002). Mature olfactory sensory neurons do not develop in mice with a targeted deletion of the achaete-scute homolog, Mash1 (Guillemot et al., 1993). Expression of Mash1 in early olfactory progenitor cells (OPCs) controls expression of the bHLH-containing transcription factors Ngn1 and NeuroD, which in turn regulate olfactory differentiation (Cau et al., 1997). The larger process of morphogenesis, patterning, and differentiation of the nasal cavity into its various sensory and nonsensory components is controlled by the spatially restricted release of various signaling molecules, such as sonic hedgehog (Shh), retinoic acid (RA), bone morphogenetic proteins (BMPs), and the fibroblast growth factor FGF8 (LaMantia et al., 2000; Kawauchi et al., 2005).

What roles, if any, are played by miRNAs during this process? We describe here the characterization of the repertoire of miRNAs expressed in the adult and the developing olfactory system, which includes several miRNA families that appear highly enriched in olfactory tissues. The specific expression of miRNA subsets by distinct olfactory cell populations in the embryo and the adult is consistent with the idea that miRNAs may play specific and significant roles in the mature and developing olfactory system. Analyses of genetically modified mice in which mature olfactory sensory neurons have been depleted of Dicer function, an enzyme required for the production of functional miRNAs (Bernstein et al., 2001), demonstrate that miRNAs are dispensable in terminally differentiated olfactory neurons. By contrast, conditional knockout of Dicer in olfactory progenitor cells causes developmental arrest and degeneration of the olfactory neuroepithelium, while the adjacent, nonneural respiratory epithelium persists. Antisense morpholino experiments in zebrafish reveal that the inhibition of expression of a single miRNA family, miR-200, largely phenocopies the defect in terminal olfactory differentiation resulting from lack of Dicer function in mouse olfactory progenitor cells. Preliminary data suggest that lunatic fringe (*lfng*) and zinc-finger homeobox 1 (*zfhx1*), two key factors associated with Notch and BMP pathways, respectively, as well as *foxg1*, a transcription factor required for normal olfactory development, may be relevant miR-200 targets. Our data support the notion that vertebrate tissue differentiation is controlled by subsets of organ-specific miRNAs.

## Results

### The Repertoire of miRNAs in the Mature and Developing Olfactory System

**Figure 1:**
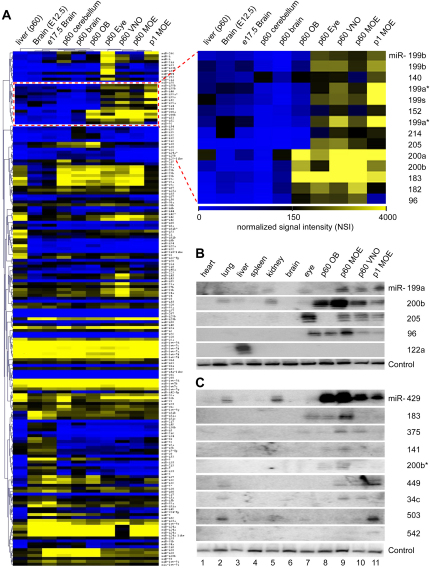
Identification of Olfactory miRNAs by Microarray and Cloning Approaches (A) Hierarchical clustering of miRNA expression profiles from several tissues using microRNA microarrays (Miska et al., 2004). The cluster of miRNAs with predicted enrichment in olfactory tissues is highlighted (right panel). Blue color indicates weak hybridization signals, and yellow indicates strong hybridization signals. miRNAs are considered present in a given tissue if they display a normalized signal intensity (NSI) ≥ 100. (B and C) Validation by northern blot analysis of miRNAs identified by microarray and cloning strategies. All tissue samples originate from adult mice (P60), excluding rat VNO (P60) and rat MOE (P1). miR-122a, known to be exclusively expressed in liver tissue, is used as a positive control. U6 snRNA serves as a loading control.

In order to understand the roles played by miRNAs during olfactory development, we aimed to identify the repertoire of miRNAs expressed in peripheral olfactory tissues. Reverse-transcribed and amplified cDNA generated from the 18–26 nucleotide small RNA fraction of olfactory as well as from various neural and nonneural tissues dissected from newborn and adult rats were hybridized to microarrays capable of detecting the expression of 138 known mammalian miRNAs (Miska et al., 2004). Ninety-four (68%) of these known miRNAs were present at detectable levels in the adult and newborn MOE, vomeronasal organ (VNO), or olfactory bulb (OB) (Figure 1A and see Table S1 available online). Forty-one miRNAs (30%), including many of the let-7 variants, show expression in all tissues examined, whether olfactory derived or not (Table S1). By contrast, we identified 12 miRNAs corresponding to 9 families (miR-199, miR-140, miR-152, miR-214, miR-205, miR-200, miR-183, miR-182, miR-96) that displayed highly enriched expression in the olfactory system (Figure 1A). Hierarchical clustering confirmed that the miRNA repertoire from each primary olfactory tissue (i.e., newborn and adult MOE and VNO) is more similar to each other than to any other neural or nonneural tissue tested. Data obtained by the microarray assay were subsequently validated by northern blot analyses (Figure 1B), which confirmed the enrichment of subsets of miRNA families in the olfactory system.

In order to comprehensively characterize the repertoire of olfactory miRNAs, including species that may not be included in the microarray described above, we systematically cloned small RNAs between 18 and 26 nucleotides in length from adult VNO and adult and newborn MOE and sequenced 3600 clones. We obtained 643, 1036, and 883 small RNAs from rat postnatal day 1 (P1) MOE tissue, P60 MOE, and P60 VNO, respectively, of which 317 (49%), 595 (57%), and 267 (30%) corresponded to known miRNAs (Table S2). Not surprisingly, miR-124 and let-7 variants, known to be highly expressed in the brain (Lagos-Quintana et al., 2002), were among the most abundant miRNAs identified by direct cloning. In addition, we cloned members of eight of the nine miRNA families predicted by the microarray assay to be highly enriched in the olfactory system. One of these families, miR-200 family comprising miR-200a, miR-200b, miR-200c, miR-429, and miR-141, also highly detected by microarray, was among the most frequently cloned species in all olfactory tissues examined (Table S2).

Excluding sequences corresponding to known miRNAs, ribosomal genes, and mRNAs, 100 small RNA sequences not present in the microarray were identified. Among them, we used the following criteria to identify genuine miRNAs: 18–24 nucleotides in length, prediction of a stem loop structure for the miRNA precursor (Zuker, 2003), and detection of an 18–24 nucleotide band by northern hybridization analyses. To distinguish miRNAs from other small RNAs or degradation products, we evaluated the probability of the ∼60 base pair genomic sequence immediately upstream and downstream of a candidate miRNA to form a hairpin structure using Mfold, a program designed for analysis of RNA secondary structure (Zuker, 2003). Thirty of the 100 clones passed the filters and were further tested for expression in olfactory tissues by northern hybridization analyses. Of these, 18 clones displayed the expected 18–24 nucleotide bands and were subsequently listed in miRBase database, among which nine appeared highly enriched in the olfactory and vomeronasal epithelia (Figure 1C).

### Cellular Distribution of microRNAs in the Mature and Developing Olfactory System

**Figure 2:**
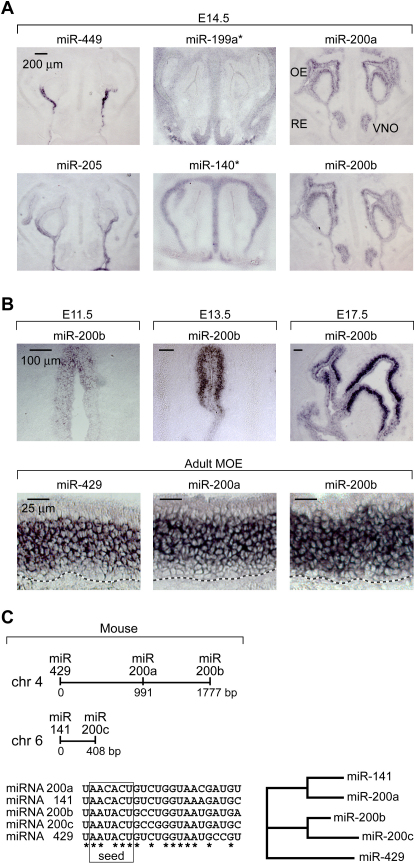
Expression Patterns of Olfactory miRNAs Analyzed by Locked Nucleic Acid-Based In Situ Hybridization (A) Three basic patterns of miRNA expression were identified during embryonic MOE development. Left: the expression of miR-34b, 34c, 139, 205, and 449 is restricted to the respiratory epithelium. Middle: the expression of miR-125b, 140^∗^, 199a, 199a^∗^, and 199b is restricted to the mesenchyme underlying or cartilage surrounding the MOE. Right: the expression of miR-96, 141, 182, 183, 200a, 200b, 191, and 429 is strongest in the MOE and VNO neuroepithelium, with reduced levels in the respiratory epithelium. OE, olfactory epithelium; RE, respiratory epithelium; VNO, vomeronasal organ. (B) Developmental time course analysis of miR-200 family member expression. (C) Genomic organization of mouse miR-200 family members.

In order to gain cellular resolution of miRNA expression, we performed in situ hybridization experiments in mouse tissues using locked-nucleic-acid (LNA)-modified DNA oligonucleotide probes (Wienholds et al., 2005; Figure 2A). Experiments in zebrafish have previously established that LNA probes specifically recognize mature miRNA species and do not hybridize with precursor miRNAs. Moreover, LNA probes are highly specific and can discriminate among members of the same miRNA family (Wienholds et al., 2005). We focused our efforts on 24 miRNAs that displayed strong and preferential expression in the developing and mature olfactory system by northern blot analyses (list, sequence, and summary of expression patterns of the 24 miRNAs are found in Table S3). Although a subset of the LNA probes (6 of 24) did not yield any signal, most probes generated detectable expression patterns. Five of 24 probes, including miR-449 and miR-205, displayed expression limited to the nonneural respiratory epithelium (Figure 2A, left column, and Table S3). Five of 24 miRNAs, including miR-199a^∗^ and miR-140^∗^ (Figure 2A, center column, and Table S3), showed expression in the mesenchyme underlying or cartilage surrounding the MOE and VNO. Finally, 8 of 24 miRNA probes, including miR-200a and miR-200b, as well as miR-96, miR-141, miR-182, miR-183, miR-191, and miR-429, revealed robust expression in the MOE and VNO neuroepithelium, with weaker expression in the adjacent respiratory epithelium (Figure 2A, right column, and Table S3). Expression was excluded from the supporting cell layer located adjacent to the nasal lumen and was detectable in both immature and mature MOE and VNO neuroepithelia (Figure 2A, right column, and 2B, lower panel). Across our study, we did not identify any miRNA species that were differentially expressed between the VNO and the MOE neuroepithelium.

The intriguing specificity and intensity of expression of the miR-200 family members in the MOE prompted us to pursue an in-depth investigation of their distribution during embryonic development and in the adult. Expression of the miR-200 family can be detected in olfactory placodes as early as E9.5, which is the first identifiable stage of olfactory development, with continued expression within the MOE anlage in the posterodorsal aspect of the olfactory pit at E11.5 (Figure 2B). From E13.5 onward, miR-200b expression becomes evenly expressed throughout the MOE at the exclusion of the supporting cell layer (Figure 2B). In the adult, the expression pattern of all miR-200 family members is restricted to the immature and mature neuronal cell layers of the MOE and is excluded from the basal and sustentacular cell layers (Figure 2B). In mouse, the miR-200 family is composed of five family members (miR-141, -200a, -200b, -200c, -429) clustered into two loci of chromosomes 4 and 6 (Figure 2C). All individual members of the miR-200 family display similar expression patterns. However, miR-141 and -200a express different 5′ seed heptamers from miR- 200b, -200c, and -429 and are thus likely to form two functional subgroups within the miR-200 family (Figure 2C; Doench and Sharp, 2004; Lewis et al., 2005). The strong, specific, and coordinated expression of miR-200 members in the MOE anlage and in the mature and immature MOE is consistent with a potential role of this miRNA family during MOE neurogenesis.

### Conditional Dicer Inactivation in Olfactory Progenitors and Mature Neurons

**Figure 3:**
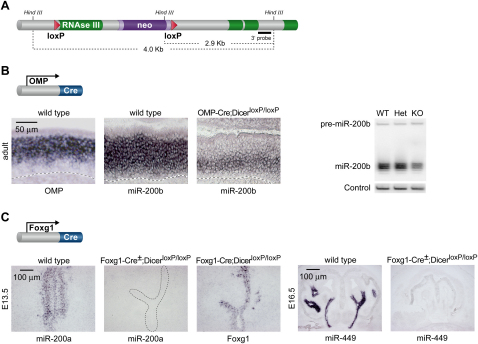
Conditional Ablation of Dicer in Mature Olfactory Neurons and Olfactory Progenitors (A) Schematic diagram of the Dicer conditional targeting construct used in this study (Harfe et al., 2005). (B) Cross of OMP-Cre and Dicer^loxP/loxP^ transgenic lines. miR-200b and OMP expression overlaps in mature neurons (left and center panels). Mature miR-200b expression is abolished in OMP-expressing cells of OMP-Cre; Dicer^loxP/loxP^ mice but remains in OMP-negative, immature neurons and progenitor cells located in the basal MOE (right panel). Broken black line indicates the basal lamina of the MOE. Northern blot analysis confirms the reduction in miR-200b expression (right blot). (C) Cross of Foxg1-Cre and Dicer^loxP/loxP^ transgenic lines. Tissues derived from the olfactory placodes of Foxg1-Cre^+/^^−^; Dicer^loxP/loxP^ tissues were analyzed for expression of mature miR-200a and miR-449 expression. Expression of Foxg1 in adjacent sections was used to demonstrate that MOE and respiratory epithelial tissue is still present in these mutants.

In order to evaluate the potential roles played by miRNAs during olfactory development and in mature olfactory neurons, we used a previously established conditional null allele of Dicer to inactivate Dicer function within specific olfactory cell types (Figure 3A; Harfe et al., 2005).

In order to abolish Dicer function in mature olfactory neurons, we took advantage of the specific expression of the olfactory marker protein (OMP) in fully differentiated MOE and VNO neurons. Mice harboring the conditional Dicer allele were crossed with a mouse line in which Cre recombinase is expressed under the control of the endogenous OMP promoter (Eggan et al., 2004). To verify the efficiency of our genetic strategy, we monitored the expression of miRNAs in OMP^+^ cells of control and mutant animals. In wild-type animals, the expression of OMP and miR-200b is partially overlapping, with OMP exclusively expressed by differentiated neurons located in the apical half of the neuroepithelium, while miR-200b is expressed throughout the neuroepithelium in both mature and immature neurons (Figure 3B). In contrast, upon Cre-mediated deletion of Dicer in OMP-positive cells, miR-200b expression is abolished from the apical portion of the neuroepithelium, while it is maintained within basal immature neurons (Figure 3B). Northern blot analysis confirmed that the level of miR-200b expression throughout the entire olfactory epithelium is reduced by ∼50%, due to the absence of miRNA processing in OMP-expressing neurons, while it remains in immature precursor cells (Figure 3B).

In order to abolish miRNA processing in olfactory progenitors, we took advantage of the early expression of Foxg1 in the developing olfactory placodes (Kawauchi et al., 2005). Mice harboring the conditional Dicer allele were crossed with a mouse line expressing Cre recombinase under the control of the endogenous Foxg1 promoter (Hebert and McConnell, 2000). Cre activity has been detected in the olfactory placodes of Foxg1-Cre mouse embryos as early as E9.5 (Kawauchi et al., 2005), ensuring that Dicer function is abolished at a stage prior to, or concurrent with, the initiation of olfactory neurogenesis. As shown in Figure 3C, miR-200a is widely expressed throughout the developing MOE neuroepithelium in embryonic day 13.5 (E13.5) wild-type mice. In marked contrast, miR-200a expression is undetectable in the MOE of E13.5 Foxg1-Cre^+/−^; Dicer^loxP/loxP^ mutants, despite the fact that the main olfactory epithelium is still present at this stage, as revealed by Foxg1 staining in adjacent sections (Figure 3C). Similarly, expression of miRNAs from the respiratory epithelium, such as miR-449, is abolished in E16.5 Foxg1-Cre^+/−^; Dicer^loxP/loxP^ mutants, confirming that Dicer function can be effectively knocked out in all structures originating from the olfactory placodes (Figure 3C). These experiments confirm that a dual genetic strategy can specifically prevent generation of mature miRNAs in olfactory neurons or in their progenitors.

### miRNAs Are Required for Maintenance but Not Initiation of Olfactory Neurogenesis

**Figure 4:**
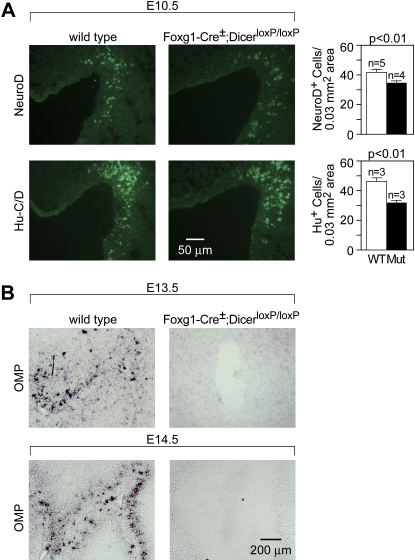
Olfactory Precursor Cells of Foxg1-Cre^+/^^−^; Dicer^loxP/loxP^ Mutants Display Normal Specification but Do Not Fully Differentiate (A) Number of differentiating and postmitotic cells in olfactory placodes was quantified by neuroD (mean ± SEM, WT 41.71 ± 2.10, n = 5; mutant 34.33 ± 1.60, n = 4, p < 0.01, Student's t test) and Hu-C/D (mean ± SEM, WT 45.82 ± 2.57, n = 3; mutant 31.32 ± 2.09, n = 3, p < 0.01, Student's t test) expression, respectively, in Foxg1-Cre^+/^^−^; Dicer^loxP/loxP^ and control E10.5 embryos. Only moderate reduction in the number of precursor cells and postmitotic neurons is observed in the mutant at this stage. Cell counts were derived from sections spanning the entire nasal pit of several animals per genotype and normalized to 0.03 mm^2^; the average MOE in a given section. (B) In situ hybridization on E13.5 olfactory epithelium fails to detect OMP expression in Foxg1-Cre^+/^^−^; Dicer^loxP/loxP^ olfactory placodes, suggesting the failure of olfactory terminal differentiation in the absence of Dicer function.

Foxg1-Cre; Dicer^loxP/loxP^ animals die in utero, have small eyes and forebrains, and develop small snouts. At E10.5, no gross morphological defect is detectable in the olfactory pits of Foxg1-Cre^+/−^; Dicer^loxP/loxP^ mutant animals relative to wild-type controls. However, the number of cells positive for neuroD, a marker of committed progenitor cells of the neuronal lineage (Cau et al., 1997), is reduced by 18% compared to mutant olfactory pits (Figure 4A, mean ± SEM, WT 41.71 ± 2.10, n = 5; mutant 34.33 ± 1.60, n = 4, p < 0.01, Student's t test). Quantification of postmitotic neurons, as assayed by Hu-C/D expression, showed a 28% reduction in olfactory pits of mutant embryos compared to wild-type controls (mean ± SEM, WT 45.82 ± 2.57, n = 3; mutant 31.32 ± 2.09, n = 3, < 0.01, Student's t test) (Figure 4A).

**Figure 5:**
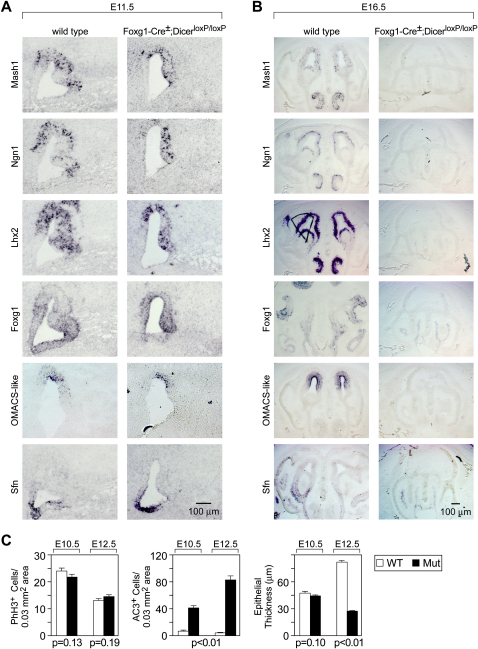
Olfactory Precursor Cells of Foxg1-Cre^+/^^−^; Dicer^loxP/loxP^ Mutants Display Normal Patterning but Do Not Fully Differentiate (A) Foxg1-Cre^+/^^−^; Dicer^loxP/loxP^ olfactory placodes at E11.5 were assayed for expression of markers that distinguish olfactory progenitor cells (Mash1, Ngn1, Lhx2, and Foxg1), MOE zonal patterning (OMACS-like), and respiratory epithelium (Sfn). Expression of these genes suggests normal gross patterning. (B) Cells of the olfactory neuronal cell lineages are lost, while nonneuronal cell lineages are maintained in Foxg1-Cre^+/^^−^;Dicer^loxP/loxP^ mutant MOE by E16.5. Expression of markers that distinguish olfactory neurogenesis (Mash1, Ngn1, Lhx2, and Foxg1) and zonal patterning (OMACS-like) cannot be detected in Foxg1-Cre^+/^^−^;Dicer^loxP/loxP^ mutant MOE at E16.5. By contrast, expression of respiratory epithelium (Sfn) persists in mutant MOE. In addition, the normally convoluted structure of the MOE is reduced to a simple epithelium comprised solely of nonneural respiratory epithelium. (C) Quantification of phospho-histone H3 and active caspase-3 immunoreactive cells in embryonic MOE of Foxg1-Cre^+/^^−^; Dicer^loxP/loxP^ mutants and controls at E10.5 (mean ± SEM, WT 23.95 ± 1.06, n = 3; mutant 21.61 ± 1.09, n = 3, p = 0.13, Student's t test) and E12.5 (mean ± SEM, WT 13.02 ± 0.76 cells, n = 3; mutant 14.49 ± 0.77 cells, n = 3, p = 0.19, Student's t test) and active caspase-3 at E10.5 (mean ± SEM, WT 7.76 ± 1.44, n = 3; mutant 41.97 ± 3.31, n = 3, p < 0.01, Student's t test) and E12.5 (mean ± SEM, WT 5.18 ± 0.54, n = 3; mutant 83.42 ± 5.54, n = 3, p < 0.01, Student's t test) indicate that loss of Dicer function results in increased cellular apoptosis and unchanged cellular proliferation in the olfactory epithelium.

By E13.5, the reduced expression of olfactory progenitor markers, such as Mash1 and Ngn1, and the marked thinning of the neuroepithelium indicate a severe defect in neurogenesis in the mutant MOE (Figure S1). Moreover, expression of mature olfactory neuronal markers, such as OMP (Figure 4B) and olfactory receptors (data not shown) is not detectable in Foxg1-Cre^+/−^; Dicer^loxP/loxP^ mutant MOE, suggesting that mutant olfactory progenitor cells do not terminally differentiate. At subsequent stages, we observe a specific loss of neuroepithelial cells that culminates in the total disappearance of markers of neuronal lineages, such as Mash1, Ngn1, Lhx2, and Foxg1 by E16.5 (Figure 5B). By contrast, development of the nonneural respiratory epithelial cells, as detected by the marker stratifin (Sfn) (Visel et al., 2004), is maintained. Thus, miRNA function appears to be required for both the terminal differentiation of olfactory neuronal precursor cells as well as for the maintenance of olfactory progenitor cells.

From early embryonic stages onward, the nasal pit is spatially segregated into several neuronal and nonneuronal components. The vomeronasal organ is located in an antero-ventral portion of the nasal septum, and the respiratory nonneuronal epithelium is located immediately ventral to the main olfactory neuroepithelium. Moreover, the MOE neuroepithelium displays a dorsoventral patterning according to which olfactory receptor gene expression is spatially restricted to one of four circumscribed zones (Ressler et al., 1993; Vassar et al., 1993). In order to evaluate whether the defect in neurogenesis described above coincides with changes in olfactory patterning, we performed in situ hybridization using markers that distinguish between the various compartments of the embryonic olfactory cavity. At E11.5, the earliest known markers of olfactory progenitor cells, Mash1 (Guillemot et al., 1993), Ngn1 (Cau et al., 1997), and Foxg1 (Kawauchi et al., 2005), as well as markers of immature neurons, such as Lhx2 (Hirota and Mombaerts, 2004) (Figure 5A and Figure S2B), show similar expression in both control and mutant animals. However, the olfactory neuroepithelium appears thinner relative to that of controls. At this stage, the expression pattern of OMACS-like, a marker of the two most dorsal MOE zones (Oka et al., 2003), is indistinguishable between wild-type and mutant MOE (Figure 5A). The zonal expression of OMACS-like is maintained at E13.5 (Figure S1).

The segregation of the nonneural respiratory epithelium from the ventral aspect of the developing main olfactory neuroepithelium was followed using Sfn as a marker. Sfn appears restricted to the ventral aspect of the developing olfactory pit at both E11.5 (Figure 5A) and E13.5 (Figure S1) in both control and mutant animals in a pattern that does not overlap with the more dorsal MOE neuroepithelium. Sfn is expressed throughout the mutant olfactory tissue at E16.5, a time point by which all neural lineages of the main olfactory neuroepithelium have degenerated and only respiratory epithelium remains (Figure 5B).

Finally, we investigated the specification of the vomeronasal placode from the medial walls of the olfactory pits and the subsequent budding of the resulting VNO toward the midline. The budding vomeronasal placode was clearly identified in both wild-type and mutant olfactory pits at E11.5, along with the expression of neurogenesis markers, such as Ngn1, Mash1, Foxg1, and Lhx2 (Figure 5A). Taken together, these results indicate that MOE cells are specified and initially maintained in Foxg1-Cre^+/−^; Dicer^loxP/loxP^ mutant MOE.

In order to determine the mechanism responsible for the reduction in olfactory neuroepithelial progenitor cells, we performed immunohistochemical analyses for both proliferating and apoptotic cells. At E10.5, the earliest stage at which a reduction in olfactory markers was observed in mutant embryos, immunostaining for the M-phase-specific marker, phosphorylated histone H3, revealed no significant changes in the number of proliferating cells between mutant and control olfactory epithelia at E10.5 (mean ± SEM, WT 23.95 ± 1.06, n = 3; mutant 21.61 ± 1.09, n = 3, p = 0.13, Student's t test), the earliest stage at which a reduction in olfactory markers was observed in mutant embryos, nor at E12.5 (mean ± SEM, WT 13.02 ± 0.76 cells, n = 3; mutant 14.49 ± 0.77 cells, n = 3, p = 0.19, Student's t test) (Figure 5C and Figure S2). By contrast, immunostaining for the apoptotic marker active caspase-3 revealed significantly increased numbers of apoptotic cells in mutant peripheral olfactory tissues at both E10.5 (mean ± SEM, WT 7.76 ± 1.44, n = 3; mutant 41.97 ± 3.31, n = 3, p < 0.01, Student's t test) and E12.5 (mean ± SEM, WT 5.18 ± 0.54, n = 3; mutant 83.42 ± 5.54, n = 3, p < 0.01, Student's t test) compared to control littermates (Figure 5C and Figure S2). Taken together, these results indicate that the loss of MOE cells is due to increased cell death rather than decreased proliferation and that, although olfactory neuroepithelial progenitor cells and their progeny are initially specified and patterned correctly in the absence of miRNA processing, they are unable to undergo terminal differentiation.

### miRNA Function Is Not Required in Mature Olfactory and Vomeronasal Neurons

In order to evaluate the contribution of miRNA functions in mature olfactory neurons, we analyzed adult OMP-Cre; Dicer^loxP/loxP^ mutant mice, in which Dicer function has been specifically abolished in fully differentiated olfactory neurons (Figure 3B). In striking contrast to the Foxg1-Cre^+/−^; Dicer^loxP/loxP^ mutants, OMP-Cre; Dicer^loxP/loxP^ mice are viable, show normal weight and survival rates, and appear to maintain normal olfactory-related functions, such as suckling, feeding, and mating.

**Figure 6:**
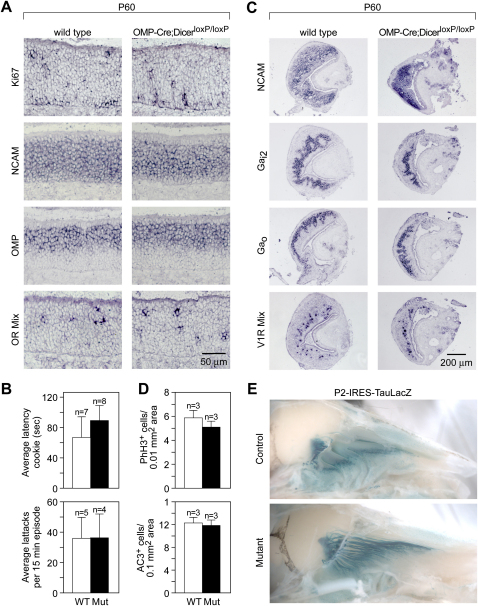
Ablation of Dicer Function in Mature Olfactory Sensory Neurons Does Not Cause Any Apparent Molecular or Behavioral Defects (A) OMP-Cre; Dicer^loxP/loxP^ adult MOE (P60) showed normal expression of molecular markers that identifies olfactory progenitor proliferation (Ki67), olfactory neuron differentiation (NCAM), and mature olfactory neurons (OMP and olfactory receptors). (B) Time required to discover a hidden cookie (latency) by OMP-Cre; Dicer^loxP/loxP^ mutant mice and control animals (mean ± SEM, WT 66.14 ± 27.91 s; mutant 88.63 ± 19.83 s; p = 0.53, Students t test) was statistically indistinguishable. Similarly, quantification of resident average attack frequency in a resident-intruder assay designed to test VNO function in OMP-Cre; Dicer^loxP/loxP^ mutants and control animals (mean ± SEM, WT 35.6 ± 13.65 s; mutant 35.75 ± 15.93 s; p = 0.99, Student's t test) showed no significant difference. (C) OMP-Cre; Dicer^loxP/loxP^ adult MOE (P60) showed normal expression of molecular markers for vomeronasal neuronal differentiation (NCAM), zonal patterning (G protein subunits) and mature function (V1 receptors). (D) Quantification of phospho-histone H3 immunoreactive cells (mean ± SEM, WT 5.79 ± 0.50, n = 3; mutant 5.05 ± 0.37, n = 3, p = 0.24, Student's t test) and active caspase-3 immunoreactive cells (mean ± SEM, WT 12.19 ± 0.77, n = 3; mutant 11.76 ± 0.74, n = 3, p = 0.69, Student's t test) in adult MOE of OMP-Cre; Dicer^loxP/loxP^ mutants and controls reveals no statistically significant differences in proliferation or apoptosis rates. (E) OMP-Cre; Dicer^loxP/loxP^; P2-IRES-TauLacZ triple-transgenic mice (P45) showed normal expression and axon targeting of LacZ in P2-expressing olfactory neurons.

We further investigated the state of the adult neuroepithelium in mutant and control animals. Cells positive for various markers of olfactory cell differentiation, such as Ki67 (Ohta and Ichimura, 2000) in dividing cells, Mash1 in basal progenitors, NCAM in immature and mature neurons, and OMP and olfactory receptors in terminally differentiated OSNs, appeared similar in wild-type and mutant MOE, both in terms of pattern and cell number (Figure 6A and data not shown). We also performed olfactory behavioral assays in order to reveal differences that may arise from the integration of multiple, subtle changes. In a crude but classic assay for olfactory function, we monitored the time required for 6- to 10-week-old control and mutant mice to locate a hidden olfactory stimulus (Stowers et al., 2002). Control animals found a hidden cookie in 66.14 ± 27.91 s compared with 88.63 ± 19.83 s for OMP-Cre; Dicer^loxP/loxP^ mutants (Figure 6B; p = 0.53, Students t test), suggesting no statistical difference in the ability to sense and respond to olfactory cues. Moreover, no statistically significant differences were observed in the rate of proliferating (mean ± SEM, WT 5.79 ± 0.50, n = 3; mutant 5.05 ± 0.37, n = 3, p = 0.24, Student's t test) or apoptotic (mean ± SEM, WT 12.19 ± 0.77, n = 3; mutant 11.76 ± 0.74, n = 3, p = 0.69, Student's t test) cells in the olfactory epithelia of mutants relative to controls (Figure 6D). Thus, we could rule out an increase in Dicer-depleted OSN apoptosis compensated by a rapid replacement of OSNs, which would have led to the absence of observable phenotypic defect in OMP-Cre; Dicer^loxP/loxP^ animals.

Similarly, we did not observe any detectable differences in marker expression between wild-type and mutant adult VNOs, including NCAM in immature and mature neurons, the V1R class of vomeronasal receptors in fully differentiated vomeronasal sensory neurons and Galpha signaling molecules that delineate zones of the VNO (Figure 6C). To test vomeronasal function, we performed a standard resident-intruder assay using 6- to 10-week-old male mice of either mutant or wild-type genetic background that had been housed in isolation for several days prior to the assay. Resident males are expected to attack a male intruder if the vomeronasal system is intact (Stowers et al., 2002). The number of aggressive attacks initiated by the resident OMP-Cre; Dicer^loxP/loxP^ mutants in every 15 min recording session appeared statistically indistinguishable from that of wild-type controls (mean ± SEM, WT 35.6 ± 13.65, n = 5; mutant 35.75 ± 15.93, n = 4, p = 0.99, Student's t test) (Figure 6B).

Olfactory (OSNs) and vomeronasal (VSNs) sensory neurons send their axons to discrete glomeruli in the main olfactory bulb (MOB) and accessory olfactory bulb (AOB), respectively. OSNs expressing a given olfactory receptor gene project their axons to two bilaterally symmetric glomeruli in the MOB (Ressler et al., 1993; Vassar et al., 1993), while VSNs expressing a given V1R or V2R receptor gene project their axons to multiple glomeruli clustered within the anterior or posterior half of the AOB, respectively (reviewed in Dulac and Torello, 2003). In order to visualize axon projections of OSNs and VSNs in the Dicer knockout background, we crossed OMP-Cre; Dicer^loxP/loxP^ mice with genetically modified mice harboring either the olfactory receptor reporter allele P2-IRES-tauLacZ (Mombaerts et al., 1996) or the V1R receptor reporter allele VN12-IRES-tauLacZ (Belluscio et al., 1999). Our data show that in the absence of miRNA function, P2-expressing OSNs and VN12-expressing VSNs are able to correctly target the appropriate glomeruli within the olfactory bulb (Figure 6E and data not shown).

Taken together, our results provide both molecular and behavioral evidence that miRNAs are largely dispensable for the function of mature olfactory and vomeronasal neurons, while they are required for olfactory differentiation in the embryo.

### An In Vivo Strategy to Block Activity of Specific miRNAs

**Figure 7:**
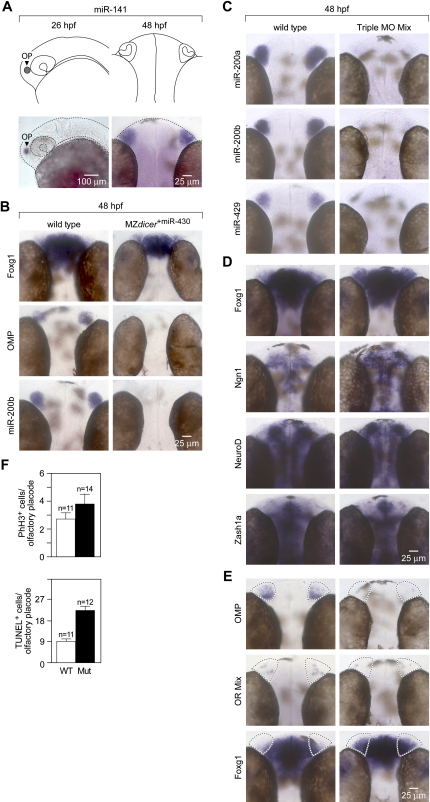
Zebrafish miR-200 Family Members Are Required for Terminal Differentiation of Olfactory Progenitor Cells (A) Schematic diagram of the zebrafish olfactory placode and olfactory organ at 26 hpf and 48 hpf, respectively, and corresponding expression pattern of miR-141, a member of the miR-200 family. (B) MZ*dicer* embryos were injected with miR-430 (MZ*dicer*^+miR-430^) to substantially rescue general neuronal and other phenotypic defects observed in MZ*dicer* mutants by supplying the critical miRNA expressed during the earliest stages of development (Giraldez et al., 2005). MZ*dicer*^+miR-430^ embryos assayed for expression of olfactory progenitor (*foxg1*), mature neuron (*OMP*), and miRNA (miR-200b) markers show impaired terminal differentiation of olfactory progenitors. (C) In situ hybridization staining of 48 hpf embryos for expression of miR-200a, miR-200b, and miR-420 that were injected at the one-cell stage with a combination of miR-141 MO, miR-200b MO, and miR-429 MO (4 ng each; Triple MO Mix) show complete loss of miR-200 family expression. (D) Wild-type and fish injected with various morpholinos at 48 hpf are morphologically indistinguishable from each other with the exception of expanded Foxg1 expression (see panel [E]). (E) Triple MO morphants injected at the one-cell stage and assayed for expression of olfactory progenitor marker (*foxg1*) and mature neuronal markers (*OMP* and an olfactory receptor mix) at 48 hpf show impaired terminal differentiation of olfactory progenitors. (F) Quantification of phospho-histone H3 immunoreactive cells (mean ± SEM, WT 2.55 ± 0.45, n = 11; morphant 3.57 ± 0.67, n = 14, p = 0.24, Student's t test) and TUNEL immunoreactive cells (mean ± SEM, WT 12.55 ± 1.46, n = 11; mutant 30.67 ± 2.59, n = 12, p < 0.01, Student's t test) in 72 hpf Triple MO morphant olfactory epithelia and controls reveals a statistically significant difference in apoptosis, but not proliferation.

Analyses of conditional Dicer mutants in the mouse reveal that miRNAs play an essential role during olfactory development. In a subsequent step, we aimed at evaluating the contribution of specific miRNA species. Determination of specific miRNA families during olfactory development in mice is difficult because genetic loss-of-function analyses are hampered by redundancy within microRNA families. We reasoned that the zebrafish could provide a useful model system due to the remarkable conservation in peripheral olfactory organization between fish and mouse at the genetic, molecular, and morphological levels (Figure 7A). For example, zonal olfactory receptor expression, signal transduction mechanisms, and olfactory bulb targeting are all conserved (reviewed in Hansen and Zielinski, 2005).

We first investigated the requirement of Dicer for zebrafish olfactory development. Removal of Dicer in maternal-zygotic *dicer* mutants eliminates all mature microRNAs during zebrafish embryogenesis and results in morphogenesis defects (Giraldez et al., 2005). Injection of miR-430 into MZ*dicer* mutants rescues early abnormalities, but does not restore the function of microRNAs that are expressed at later stages of development. We therefore analyzed olfactory development in MZ*dicer* mutants injected with miR-430 microRNAs. Early patterning of the nervous system is unperturbed in MZ*dicer*^+miR430^ mutants, e.g., markers for specified optic stalk, forebrain, midbrain-hindbrain boundary, otic vesicles, hindbrain rhombomeres, dorsal neural tube, and ventral neural tube are present (Giraldez et al., 2005). However, in contrast to control animals, the expression of markers of terminally differentiated olfactory sensory neurons, such as OMP and olfactory receptors, is largely abolished in MZ*dicer*^+miR-430^ mutants at 48 hpf (Figure 7B). In addition, the expression of *foxg1*, a marker for early olfactory stages in mice (Kawauchi et al., 2005 and Figure S2B), is upregulated, suggesting an expansion of olfactory progenitors that might be unable to mature into OSNs in absence of microRNAs (Figure 7B). These results indicate that miRNAs are critical for normal olfactory neurogenesis in both zebrafish and mouse.

To evaluate the contribution of specific miRNAs, we focused on the miR-200 family, which is highly and specifically expressed in the developing olfactory system. The function of miR-200 during olfactory development is likely to be conserved throughout evolution, as judged from the absolute conservation of miR-200 orthologs between mouse and zebrafish with respect to the relative genomic clustering position, the conserved seed region sequences, the conserved size of the family, and the conserved arm of the hairpin that generates the mature miRNA (Figure S3). Moreover, as shown in the mouse, miR-200 family members display early expression in zebrafish (Wienholds et al., 2005) and appear highly enriched in olfactory tissues by the time olfactory placodes arise at 26 hpf (Figure 7A). Antisense morpholino oligonucleotides complementary to microRNAs hairpin sequences have been shown to specifically abolish mature miRNA activity (Flynt et al., 2007; Kloosterman et al., 2007). We designed three morpholino antisense oligonucleotides predicted to each target the mature sequence of one or a few members of the miR-200 family (Figure S4A). The morpholino sequences lacked any homology to other known zebrafish transcripts. To identify the minimal concentration at which the morpholinos used in our experiments can inhibit the generation of cognate miRNAs, we injected one-cell zebrafish embryos with a range of concentrations (1 ng to 6 ng per embryo) and incubated the morphants from 18 hpf to 48 hpf before analysis. In situ hybridization analyses using LNA antisense probes to detect mature miRNAs indicated that 4 ng per embryo per miR-200 family member was the minimal dose required to knock down miRNA expression to threshold levels of detection (data not shown). Consequently, we used 4 ng dosages in all proceeding experiments. In order to test the specificity of each morpholino (MO) sequence, we systematically injected one-cell zebrafish embryos with either miR-141 MO, miR-200b MO, or miR-429 MO and performed in situ hybridization against all five miRNAs of the miR-200 family. As predicted from sequence analyses and thermal stability calculations, miR-141 MO specifically inhibited miR-200a and miR-141, miR-200b MO specifically inhibited miR-200b and miR-200c, and miR-429 MO specifically inhibited miR-429 (Figure S4B). In addition, in situ hybridization analyses (Figure 7C) show that a mixture of all three morpholinos (Triple MO mix: miR-141 MO, miR-200b MO, and miR-429 MO) was sufficient to simultaneously inhibit the expression of all five mature zebrafish miR-200 family members to threshold levels of detection.

Antisense experiments can be plagued by nonspecific phenotypes, such as cell death in the head, general neural degeneration, CNS necrosis, and general lethality, which are likely to result from nonspecific interactions of MOs with inappropriate targets (reviewed in Sumanas and Larson, 2002). In order to test for such effects in our experiments, we performed in situ hybridization with a number of genes widely expressed in the nervous system. Our data show that expression patterns of genes expressed throughout the brain and in areas devoid of miR-200 family expression were comparable between wild-type and triple MO morphants, indicating that widespread neural defects were absent in the morphant fish (Figure 7D). Furthermore, analyses of wild-type fish and fish injected with individual or mixtures of MOs did not display any morphological signs of widespread cell death, necrosis, or lethality (Figure S4C). We conclude that mature zebrafish miR-200 family members can be specifically and efficiently knocked down in various combinations in the developing olfactory system using antisense morpholinos without confounding “off-target” effects.

### miR-200 Family Members Are Required for the Proper Differentiation of Olfactory Progenitor Cells

Embryos injected with individual antisense morpholinos showed knockdown of the expected miRNAs but did not display any visible olfactory phenotype, as visualized by a normal pattern of OMP expression in morphant fish (data not shown). We next wished to determine whether the distinct 5′ seeds contributed differentially to the physiological function of the miRNA-200 family. Embryos injected with either miR-141/miR-200a or miR-200b/miR-429 pairs of antisense morpholinos showed lack of expression of the corresponding miR-200 members with a given 5′ seed but did not display any change in OMP expression relative to wild-type controls (data not shown). Finally, we eliminated the function of all miR-200 family members by injecting embryos simultaneously with the Triple MO mix. Forty-eight hours after injection, triple MO morphants showed a reduction of OMP and olfactory receptor expression in the developing olfactory epithelium relative to wild-type controls (Figure 7E). We also observed a concomitant increase in *foxg1* expression in the presumptive area of the olfactory epithelium (Figure 7E). These results indicate that the functional loss of the miR-200 family precludes normal differentiation of olfactory progenitor cells into mature olfactory neurons and thus phenocopies an important aspect of the Dicer knockout phenotype observed both in mice and zebrafish.

We subsequently performed immunohistochemical identification of proliferating and apoptotic cells in order to determine whether miR-200 morphant olfactory phenotypes are accompanied by increased cellular apoptosis, as observed in Dicer null mouse olfactory placodes. Immunostaining for the M-phase-specific marker, phosphorylated histone H3, at 72 hpf revealed no significant changes in the number of proliferating cells between mutant and control olfactory epithelia (mean ± SEM, WT 2.55 ± 0.45, n = 11; morphant 3.57 ± 0.67, n = 14, p = 0.24, Student's t test) (Figure 7F). By contrast, miR-200 morphant olfactory epithelia presented significantly increased numbers of apoptotic cells relative to wild-type controls (mean ± SEM, WT 12.55 ± 1.46, n = 11; mutant 30.67 ± 2.59, n = 12, p < 0.01, Student's t test) (Figure 7F), as detected by TUNEL staining. Taken together, these results indicate that in the absence of miR-200 family expression during olfactory placodal development, zebrafish olfactory progenitors are unable to undergo normal terminal differentiation and, instead, undergo apoptosis. This phenotype closely resembles the olfactory defect resulting from the lack of Dicer expression by mouse olfactory progenitors.

### Notch and TGFβ Signaling Pathways and Foxg1 Are Candidate Targets of the miR-200 Family

**Figure 8:**
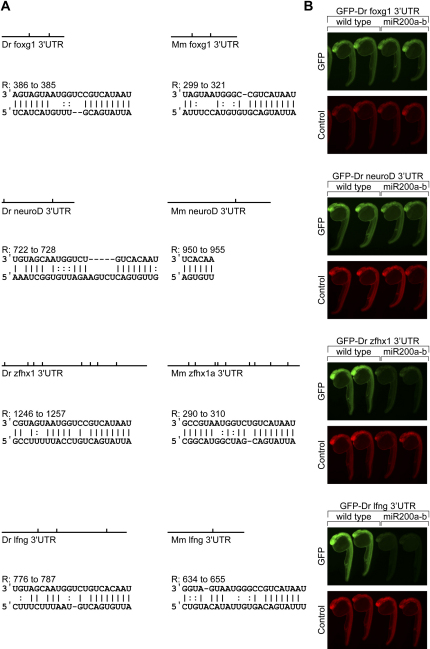
miR-200 Target Validation (A) Comparison of conserved miR-200 sites in the 3′UTRs of select miR-200 predicted targets in mouse and zebrafish suggest that miR-200 family members may be sufficient to negatively regulate *zfhx1*, *foxg1*, and *lfng* and may help to downregulate *neuroD*. Vertical ticks on schematic drawings indicate a predicted miR-200 site, and the alignments correspond to the strongest miR-200 site produced by the miRanda algorithm (Enright et al., 2003). (B) GFP reporters fused upstream of full-length zebrafish 3′UTRs corresponding to putative targets containing predicted miR-200 binding sites were coinjected with control DsRed mRNA into wild-type zebrafish embryos at the one-cell stage either in the absence or presence of synthetic miR-200a/miR-200b RNA duplex. Fluorescent microscopy shows GFP reporter expression (green) and control DsRed expression (red) at 25–30 hpf, indicating that miR-200 family members are sufficient to downregulate zebrafish *zfhx1* and *lfng*.

To gain further insights into the role of the miR-200 family in mediating olfactory differentiation, we used a bioinformatic approach to predict and validate potential miR-200 targets. We used the web-accessible miRNA target prediction algorithm, miRanda, which was capable of conveniently analyzing zebrafish 3′UTRs at the time of inquiry (Enright et al., 2003). The olfactory phenotype observed in both Foxg1-Cre; Dicer^loxP/loxP^ mice and morphant fish prompted us to focus our attention on targets with known roles in the regulation of neuronal differentiation, and in particular on four genes: *neuroD* and *foxg1*, genes required for olfactory progenitor cell differentiation in mice, ranked in the top 40 and 220 hits out of 736 total hits, respectively (data not shown); and *lfng*, a modifier of the Notch signaling pathway; and *zfhx1*, an enhancer of TGFβ signaling, located within the top 20 hits. These genes are expressed in the basal cell layer and lamina propria of mouse MOE, respectively, and are associated with Notch and BMP signaling pathways shown to be essential for mouse MOE development (Beites et al., 2005; Cau et al., 2002). Due to the molecular and cellular similarity of mouse and zebrafish olfactory development processes and the high degree of conservation between the miR-200 miRNAs in the respective organisms, we reasoned that physiologically meaningful targets were likely to be conserved between the zebrafish and mouse genomes. We used the MicroCosm system that interfaces the miRanda prediction software with miRBase, the accepted database of miRNA classification, to confirm that mouse orthologs of zebrafish *neuroD*, *foxg1*, *zfhx1*, and *lfng* have conserved miR-200 seeds in their 3′UTRs (Griffiths-Jones et al., 2006) (Figure 8A). MicroRNA binding sites containing homology to 5′ seeds (8-mer, positions 1–8) represent the best indicator of likely miRNA targets (Grimson et al., 2007), and this arrangement applies to all four predicted targets in zebrafish (Figure 8A). In addition, the mouse orthologs of *foxg1* and *zfhx1* maintain strong 8-mer 5′ seeds while homology to 5′ seed heptamers (7-mer, position 2–8) also yields high signal-to-noise predictions in the mouse lfng 3′UTR (Lewis et al., 2005) (Figure 8A). Moreover, increased *foxg1* expression observed in the zebrafish morpholino experiments and in the mouse conditional Dicer microarray experiments (Table S3) also suggests that *foxg1* may be a genuine miR-200 family target. We conclude that *foxg1*, *zfhx1*, and *lfng* are likely to be genuine targets for miR-200 family members in both mouse and zebrafish olfactory systems, while *neuroD* might only be a target in the fish.

In order to further validate the physiological requirement for miR-200's action on these targets, we generated GFP reporters containing the full-length 3′ UTRs for zebrafish *neuroD*, *foxg1*, *zfhx1*, and *lfng* (Giraldez et al., 2006). Exogenous miR-200 duplex RNA was able to reduce expression of the *lfng* and *zfhx1* reporters, while miR-200 duplexes did not affect GFP expression levels for the *foxg1* and *neuroD* reporters (Figure 8B). These results argue that *lfng* and *zfhx1* can be efficiently downregulated by the miR-200 family alone, whereas *foxg1* and *neuroD*, although likely genuine targets, may require the combined action of several miRNA species in addition to miR-200 action, in order to be efficiently downregulated.

## Discussion

The exact roles played by miRNAs during biological processes and the precise mechanisms by which they exert a regulatory function are currently under intense experimental scrutiny. Potential regulatory functions of miRNAs in the developing and adult nervous system are particularly intriguing. For example, more than half of the 115 zebrafish miRNAs for which spatial and temporal expression patterns were obtained exhibited expression in specific regions of the central nervous system (Wienholds et al., 2005), and the key contribution of miRNAs in invertebrate neurogenesis may suggest similar roles during vertebrate neural development (Kosik and Krichevsky, 2006; Cao et al., 2006). Our study took advantage of the molecular and genetic amenability of the olfactory system to gain insights into the specific contribution of miRNA-mediated regulation in vertebrate neurogenesis and in neuronal function.

We first aimed at identifying the repertoire of miRNAs expressed by olfactory sensory neurons and by their embryonic progenitors. From the over 100 distinct miRNAs identified in olfactory tissues, the most abundant miRNAs isolated from our study include species that are widely expressed in many neural tissues (miR-124a and let-7 variants), as well as a highly restricted family of miRNAs (miR-200). Subsequent northern and in situ hybridization analyses confirmed that around 20 miRNA species are enriched in olfactory tissues.

In order to determine whether miRNAs are required during olfactory neuronal development, we analyzed embryonic tissues in which Dicer function had been specifically ablated in olfactory progenitor cells. Our data show that loss of miRNA function from olfactory progenitor cells produced no detectable alterations in patterning, such as main olfactory epithelial zonal patterning, initial cell fate specification, or induction of nonneural respiratory epithelium. Similarly, loss of Dicer function in several other developing tissues has been shown to leave early patterning events relatively unperturbed. For example, conditional Dicer ablation in skin epithelial progenitors does not preclude initial perinatal epidermal cell differentiation, and loss of Dicer function in developing limb mesoderm does not affect digit number or cartilage patterning (Andl et al., 2006; Harfe et al., 2005; Yi et al., 2006). In contrast, we find that terminal differentiation of the olfactory progenitor pool into mature olfactory neurons does not occur and that the olfactory precursor cell population is not maintained. In addition, the MOE epithelial cells selectively degenerate due to increased apoptosis, while the nonneural respiratory epithelium appears to develop relatively unperturbed despite the loss of miRNA function in these cells. This supports the idea that phenotypes resulting from conditional Dicer ablation are mostly manifested during the terminal differentiation phase of progenitor development. Accordingly, in the absence of miRNA activity, skin epidermal cells have been shown to develop into deformed cysts rather than invaginating, and limb buds undergo growth arrest due to global apoptosis (Andl et al., 2006; Harfe et al., 2005; Yi et al., 2006). It is also unlikely that the observed phenotypes are due to non-cell-autonomous effects (e.g., defects in olfactory bulb-derived signals) because respiratory epithelial identity is maintained and OSNs are able to terminally differentiate despite the complete absence of an olfactory bulb (Sullivan et al., 1995). Although recent reports suggest the possibility that Cre recombinase toxicity may at least in part be responsible for the observed increase in cell death (Lee et al. 2006; Schmidt-Supprian and Rajewsky, 2007), this reason is unlikely to be the cause of the observed apoptosis phenotype, given that no perturbations were observed in either Foxg1-Cre^+/−^; Dicer^+/loxP^ control animals, which are viable, or OMP-Cre; Dicer animals in which OMP represents 0.5% of the total RNA per olfactory neuron (Rogers et al., 1985).

A unique aspect of our study was the phenotypic comparison of conditional Dicer ablation at two different stages of olfactory development—olfactory progenitor cells and terminally differentiated olfactory sensory neurons. In marked contrast to the severe phenotype observed in Foxg1-Cre; Dicer^loxP/loxP^ olfactory placodes, specific ablation of Dicer function in mature olfactory neurons produced no observable abnormal phenotype, as assessed by molecular, behavioral, and axon guidance assays. Although miRNA-mediated regulation has been proposed to be physiologically relevant to mature neuron function (Schratt et al., 2006), our results suggest that miRNA activity in mature olfactory neurons is dispensable in vivo.

We next addressed the issue of the specific contribution of discrete miRNA species in mediating olfactory development. It is widely assumed that miRNA redundancy may greatly challenge the analysis of specific miRNA function (Plasterk, 2006). Indeed, very few miRNA mutants have been identified in traditional forward screens using such genetically tractable systems as the fruit fly *Drosophila* or the nematode *C. elegans*. Successful identification of individual miRNA functions has been accomplished in experimental systems in which the miRNA species of interest constituted a substantial fraction of the total miRNA population (Giraldez et al., 2005; Zhao et al., 2005). Accordingly, we decided to focus our efforts on potential functions mediated by the miR-200 family, which is among the most highly and most specifically miRNA subset expressed in the developing olfactory system. The similarity in the cellular and molecular process of olfactory development in zebrafish and mouse and the parallel olfactory defects observed in MZ*dicer*^+miR-430^ zebrafish embryos and in Foxg1-Cre; Dicer^loxP/loxP^ mouse embryos allowed us to use an antisense morpholino-mediated strategy (Flynt et al., 2007; Kloosterman et al., 2007). Knocking down the expression of mature miR-200 family members led to impairment of mature olfactory marker expression and expansion of the early marker, foxg1, in the olfactory primordium. These results suggest that the loss of miR-200 family function disrupts terminal differentiation of olfactory progenitor cells, thus phenocopying an important aspect of the defects observed in mouse Foxg1-Cre; Dicer^loxP/loxP^ mutant MOE. The miR-200 family is therefore among the first neuronal miRNA families in vertebrates with a loss-of-function phenotype.

How does the miR-200 family mediate its control of olfactory neurogenesis? Intriguingly, miR-200 family members are coordinately expressed from different loci, yet members express different 5′ seed heptamers, changes in which are thought to alter the binding specificity to target mRNA (Doench and Sharp, 2004; Lewis et al., 2005). The morpholino knockdown experiments show that miR-200 family members are likely to act redundantly, even though they display different 5′ seed regions. In addition, our preliminary microarray and GFP-sensor experiments suggest that *foxg1* itself, as well as lunatic fringe (lfng) and zinc-finger homeobox 1 (zfhx1), two key factors associated with Notch and BMP pathways, respectively, may be genuine miR-200 targets. Further experiments must be conducted to determine the physiological relevance of these targets. In addition, other predicted miR-200 family targets may also contribute to the olfactory phenotypes observed in morphant fish and the Foxg1-Cre;Dicer^loxP/loxP^ mutant mice.

Recently, independent reports have demonstrated that the miR-200 family is highly expressed in skin epidermal cells (Yi et al., 2006). The progenitors of this epidermal cell population are thought to share many common mechanisms of progenitor cell development with olfactory progenitors. For example, cytokeratin 14, a marker of skin epithelial progenitor cells, is also expressed in olfactory basal progenitors (Holbrook et al., 1995; Vaioukhin et al., 1999), and both cell types regenerate throughout life. Moreover, lfng is expressed in the basal layer of the epidermis containing the progenitor cells (Thélu et al., 1998), and both Notch and BMP signaling are important regulators of epidermal progenitor differentiation (Botchkarev and Sharov, 2004; Nicolas et al., 2003). Thus, the regulatory step involving the miR-200 family, and shown here to be essential for olfactory neurogenesis, may be employed by other systems of epithelial origin to ensure the proper mediation of critical signaling cascades during development.

## Experimental Procedures

### miRNA Isolation, miRNA Microarray, and Small RNA Cloning

Total RNA was isolated as described in Supplemental Data. 300 μg of total RNAs for each tissue were size fractionated on denaturing PAGE gels. MiRNA printing was exactly as described previously (Miska et al., 2004), and microarrays were hybridized and analyzed as described in Supplemental Data. Small RNA cloning experiments were conducted in a similar manner as described in Supplemental Data. Expression of identified miRNAs was confirmed by northern hybridization analyses as described in Supplemental data.

### Immunostaining and Cell Counting

Immunostaining and cell counting of mouse tissues were performed as described in Supplemental Data using the following primary antibodies: sc-1084 (1:500, anti-neuroD, Santa Cruz Biotech), anti-Hu-C/D (1:200, Molecular Probes), anti-phospho-histone H3 (PH3, 1:200, Upstate Biotechnology), and rabbit anti-active-caspase-3 (AC3, 1:250, Promega) primary antibody.

Immunostaining and cell counting of zebrafish tissues were performed as described in Supplemental Data using anti-HuC (1:1000, Molecular Probes) in combination with either anti-PH3 (1:500, Upstate Biotechnology) or TUNEL staining using the Apoptag Fluorescein Apoptosis Detection Kit (Chemicon).

### In Situ Hybridization and RNA Probes

LNA probes were purchased from Exiqon SA, labeled using a DIG 3′ end labeling kit (Roche), and purified using Sephadex G25 MicroSpin columns (Amersham). Whole-mount in situ hybridizations were performed essentially as described in Schier et al. (1997) and Wienholds et al. (2005). RNA in situ hybridization on mouse sections was performed as described (Schaeren-Wiemers and Gerfin-Moser, 1993). MOE tissue was dissected and freshly frozen in Tissue-Tek OCT compound (Sakura Finetek).

### Olfactory Behavior and Resident-Intruder Assays

The time required for 6- to 10-week-old mice to unearth an olfactory stimulus (cookie) hidden within the pine bedding of a large cage at the opposite corner was measured. The resident-intruder assay was performed essentially as described in Stowers et al. (2002). Behaviors from both assays were recorded using Protech video equipment and software.

### Zebrafish Microinjection Experiments

Morpholinos targeting the miR-200 family were generated as described in Supplemental Data. Morpholinos, either alone or in combination, were diluted in phenol red to a final concentration of 2 ng/nl each. For 3′UTR sensor assays, 3′UTR sensor constructs were generated as described in Supplemental Data and were microinjected into one-cell zebrafish embryos according to the methods described in Supplemental Data.

## Supplemental Data

Document S1. Supplemental Experimental Procedures and Figures
