# Supplementary material for: Members of the miRNA-200 Family Regulate Olfactory Neurogenesis
Source: Neuron. 2008 Jan 10;57(1):41–55. doi: 10.1016/j.neuron.2007.11.018 (PMC2204047; doi:10.1016/j.neuron.2007.11.018)
Supplement: Document S1. Supplemental Experimental Procedures and Figures [file mmc1.pdf]

**Neuron, Volume *57***

# Supplemental Data

## Members of the miRNA-200 Family Regulate Olfactory Neurogenesis

Philip S. Choi, Lisa Zakhary, Wen-Yee Choi, Sophie Caron, Ezequiel Alvarez-Saavedra, Eric A. Miska, Mike McManus, Brian Harfe, Antonio Giraldez, Robert H. Horvitz, Alexander F. Schier, and Catherine Dulac

## Supplemental Experimental Procedures

*Total RNA Isolation*

For miRNA cloning and microarray experiments, Sprague Dawley rat P1 MOE and P60 main olfactory epithelium (MOE), vomeronasal epithelium (VNO), olfactory bulb (OB), cerebellum, brain, eye and liver were dissected and immediately frozen on dry ice. For northern hybridization, CD-1 mouse P60 heart, lung, liver, spleen, kidney, brain, eye, OB, MOE, rat P60 VNO and rat P1 MOE tissues were used. Tissues from 8-13 animals were pooled. Frozen tissues were placed in Trizol (Invitrogen) and RNA isolation was performed according to manufacturer's instructions.

*miRNA Isolation, miRNA Microarray and Small RNA Cloning*

18-26-nucleotide RNAs were identified using radiolabeled 18-mer (5'-AGCGUGUAGGGAUCCAAA-3') and 26-mer RNA (5'-GGCCAACGUUCUCAACAAUAGUGAGA-3') oligos. 3' and 5' adapter oligos were ligated to the purified 18-26-nucleotide RNAs (3' adapter, 5'-UUUaaccgcgaattccagt-3'; 5' adapter, 5'-acggaattcctcactAAA-3', were X=RNA and x=DNA). The ligation products were reverse transcribed with 5'-GACTAGCTGGAATTCGCGGTTAAA-3' in 10 cycles (94°C, 40 sec; 50°C, 30 sec; 72°C, 30 sec). For the microarray, subsequent cDNA

samples were amplified using the following primers: 5'-Cy3-ACGGAATTCCTCACTAAA-3' and 5'-TACTGGAATTCGCGGTTAA-3'. PCR products were precipitated and resuspended in hybridization buffer (5x SSC, 0.1% SDS and 0.1 mg/mL salmon sperm DNA). MiRNA printing was exactly as described previously (Miska et al., 2004) and microarrays were hybridized and analyzed as described in Supplemental Data.

For small RNA cloning experiments, cDNA samples were amplified with 5'-GACTAGCTGGAATTCGCGGTTAAA-3' and 5'-CAGCCAACGGAATTCCTCACTAAA-3'. PCR products were purified with phenol/chloroform extraction and digested with EcoR-I. Digested samples were concatemerized with T4 DNA ligase and run on a 2% GTG Nusieve agarose gel. A smear corresponding to 300-500 bp concatamers was excised and purified. Following a Taq fill-in reaction, samples were cloned into pCRII-TOPO Vector (Invitrogen), plated and sequenced. High-quality sequences were subjected to local BLASTN analyses against the 3518 annotated miRNA sequences in miRBase sequence database release 8.0 (Sanger Center, Feb 2006) to identify known miRNA sequences. Unknown sequences were subjected to NCBI BLAST analyses.

*Northern Hybridization*

30 ug of total RNAs from each tissue was size fractionated on a 15% denaturing polyacrylamide gel and transferred to a Genescreen Plus nylon membrane (NEN) at 3.3 $mA/cm^2$ for 40 minutes. Membranes were UV cross-linked (Stratalinker) followed by a 1 hr. incubation at 80°C. Radiolabeled probes (see below) were hybridized to blots

overnight at 50°C in a rotating hybridization oven in the following solution: 5x SSC, 20 mM $Na_2HPO_4$ (pH 7.2), 7% SDS, 2x Denhardt's solution and 0.04 mg/mL sheared salmon sperm DNA. Blots were washed four times (2x at 10 min. each then 2x at 20 min. each) at 50°C with 3x SSC, 25 mM $Na_2HPO_4$ (pH 7.2), 5% SDS and 10x Denhardt's solution. Blots were then washed in 1x SSC, 1% SDS for 5 min. at 50°C, exposed to a phosphoimager screen for 1-2 days, imaged using a Storm Imager (Molecular Dynamics), and visualized using ImageQuant software (Molecular Dynanmics). Blots were stripped by immersing in water for five minutes and reprobed up to three more times. Northern probes were generated from 10 uM oligos in a 20 uL reaction volume end-labeled with $^{32}$P-γ-ATP (ICN, 7,000 Ci/mMole) for 1 hour at 37°C. Probes were purified using G-25 MicroSpin columns (Amersham) and roughly half of the recovered volume was used for each northern assay. The probe sequences were the following: miR-199a (5'-GAACAGGTAGTCTGAACACTGGG-3'), miR-200b (5'-GTCATCATTACCAGGCAGTATTA-3'), miR-205 (5'-CAGACTCCGGTGGAATGAAGGA-3'), miR-96 (5'-AGCAAAAATGTGCTAGTGCCAAA-3'), miR-122a (5'-TGGAGTGTGACAATGGTGTTTGT-3'), miR-429 (5'-CGGCATTACCAGACAGTATTA-3'), miR-183 (5'-TTTATGGCCCTTCGGTAATTCA-3'), miR-375 (5'-TCACGCGAGCCGAACGAACAAA-3'), miR-141 (5'-ACACTGCACTGGAAGATGGA-3'), miR-449 (5'-ACCAGCTAACAATACACTGCCA-3'), miR-34c (5'-CCTGGCTGTGTGGTTAGTGAT-3'), miR-503 (5'-

CTGCAGTACTGTTCCCGCTGCTA-3’), miR-542 (5’-TCGTGACATGATGATCCCCGAG-3’) and U6 snRNA loading control (5’-TGTGCTGCCGAAGCGAGCAC-3’)

*Immunostaining and Cell Counting*

For mouse tissues, cryosections were fixed in 4% paraformaldehyde at room temperature (RT) for 25 min., permeablized in 0.2% Triton X-100/PBS for 5 min. at RT, blocked with 10% normal serum in PBS for 2 hrs. at RT, incubated for 2 hrs. at RT with primary antibody diluted in blocking solution, probed with secondary antibodies (i.e. Alexa488-conjugated donkey-anti rabbit, anti-goat or anti-mouse secondary antibodies) (1:300, Molecular Probes) for 2 hrs. at RT, stained with Hoechst 33342 (1:400, Molecular Probes), and mounted in Aqua Polymount (Polysciences, Inc.) with appropriate washes in PBS for 5-10 mins. between steps. A minimum of three animals (typically three to six) were used per group. For each animal, sections spanning the olfactory epithelium of a given developmental timepoint were stained and imaged using a Leica DMR microscope (Leica). ImageJ software (NIH) was used to count cells, calculate the area and determine the average epithelial thickness for both left and right halves of the MOE in each section.

Immunostaining of OMP-Cre;Dicer MOE cryosections was performed as above, except that isolated MOE tissue was isolated from age matched animals between 7 and 10 weeks of age, fixed in 4% paramformaldehyde for 1 hr. at RT, and cryoprotected in 30% sucrose prior to embedding. $PH3^+$ and $AC3^+$ cells were counted at three random ectoturbinate locations for each section throughout the rostrocaudal extent of the nasal cavity.

Immunostaining and cell counting of zebrafish tissues for HuC and PH3 or TUNEL were performed as follows. 72 hpf triple MO morphant or control embryos were fixed in 4% paraformaldehyde at RT, rehydrated through 75%, 50% and 25% MeOH in PBS for 5 min. each, digested with 30 ug/mL proteinaseK for 45 min., washed 4x for 30 min. each in PBS/0.1% Tween-20 (PBT), blocked for 1 hr. at RT in PBT with 10% sheep serum (blocking solution), and incubated overnight at 4°C with anti-HuC (1:1000, Molecular Probes) in order to delineate the olfactory epithelia. Embryos were then washed 4x for 30 min. each in PBT, blocked for 1 hr. at RT in blocking solution, and the anti-HuC signal was detected using the Vectastain ABC (Vector Laboratories) and TSA Cyanine 3 Tyramide (Perkin-Elmer) kits according to the manufacturer's instructions. After HuC staining, embryos were washed 3x in PBT for 5 min. each, blocked for 1 hr. at RT in blocking solution, and incubated with anti-PH3 (1:500, Upstate Biotechnology) antibody diluted in blocking solution overnight at 4°C. Embryos were then washed 4x for 30 min. each in PBT, blocked for 1 hr. at RT, and then probed with Alexa488-conjugated donkey-anti rabbit secondary antibody (1:250, Molecular Probes). Alternatively, embryos stained for HuC were stained for TUNEL using the Apoptag Fluorescein Apoptosis Detection Kit (Chemicon) with volumes adapted for staining of zebrafish embryos. Embryos were washed 3x for 5 min. each in PBT, mounted frontally in 0.2% agarose and imaged on a Zeiss Pascal confocal microscope using a 25x water immersion lens. Statistical analyses of cell counts were performed as described above.

*Morpholino Sequences*

Morpholinos targeting the miR-200 family comprise the following sequences: Anti-miR-200a (5'-AACATCGTTACCAGACAGTGTTAGA-3'), anti-miR-141 (5'-GCATCGTTACCAGACAGTGTTA-3'), anti-miR-200b (5'-GTCATCATTACCAGGCAGTATTA-3') and anti-miR-429 (5'-ACGGCATTACCAGACAGTATTA-3'). Morpholino oligos were obtained from GeneTools and dissolved in water at a concentration of 10 mg/mL.

*3' UTR Sensor Assays*

Full length 3'UTRs were cloned downstream of the GFP coding sequence in the pCS2+GFP vector by insertion into the XhoI and XbaI restriction sites. The 5' regions of miR-430 sites found within zebrafish *lfng* and *zfhx1* 3'UTR sequences were mutated by site-directed mutagenesis according to Giraldez et al., 2006. mRNA synthesis, miRNA duplexes and *in vivo* injections were performed according to Giraldez et al., 2006. The following miRNA duplex sequences were obtained from IDT: miR-200a duplex: 5'-UAACACUGUCUGGUAACGAdTdG-3' and 5'-UCGUUACCAGACAGUGUAAdTdT-3' and miR-200b duplex: 5'-UAAUACUGCCUGGUAAUGAdTdG-3' and 5'-UCAUUACCAGGCAGUAUAAdTdT-3'. Wild-type, 1-cell zebrafish embryos were injected with the respective GFP-reporter mRNA and dsRed control mRNA and a subset was further injected with 500-1000 pL of a solution containing miR-200a and miR-200b duplex each at 10 uM. Embryos injected with or without the miRNA duplex were photographed side by side at 25-30 hours after injection using fluorescent microscopy.

*Mouse and Zebrafish Lines*

The conditional Dicer, Foxg1-Cre, OMP-Cre, P2-IRES-tauLacZ, VN12-IRES-tauLacZ lines were previously described and PCR genotyped accordingly (Harfe et al., 2005; Hebert and McConnell, 2001; Eggan et al., 2004; Mombaerts et al., 1996; Belluscio et al, 1999). MZ*dicer* fish were generated as described (Giraldez et al., 2005). MZ*dicer* mutant embryos carried the $dicer^{hu715}$ or $dicer^{hu896}$ *alleles.*

## Supplemental Figures

**Supplementary Figure 1. Foxg1-Cre$^{+/-}$;Dicer$^{loxP/loxP}$ Mutant Olfactory Neuroepithelium is Disrupted**

By E13.5, the expression of olfactory progenitor differentiation markers is significantly reduced in mutant MOE relative to wild type controls. However, the presence of zonal patterning (OMACS-like) and respiratory epithelium (Sfn) markers indicates that MOE patterning remains intact in mutant MOE.

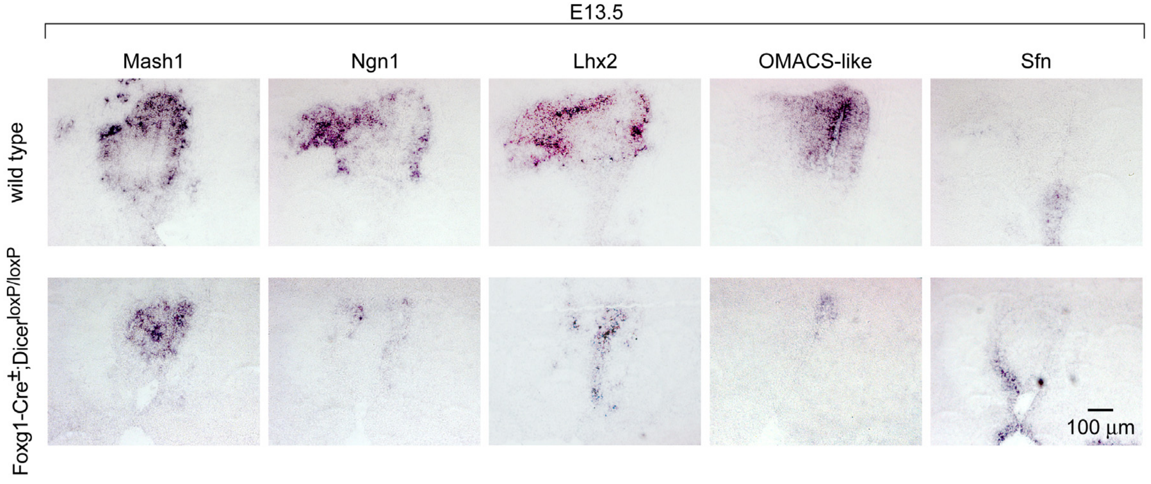

**Supplementary Figure 2. Dicer and Foxg1 Function are Required For Cell Survival During MOE Neurogenesis**

(A) Confocal images of phospho-histone H3 and active caspase-3 immunoreactive cells in embryonic MOE of Foxg1-$Cre^{+/-}$; $Dicer^{loxP/loxP}$ mutants and controls indicate that loss of Dicer function results in increased cellular apoptosis and unchanged cellular proliferation in the olfactory epithelium. Broken white line indicates the basal lamina of the MOE.

(B) In situ hybridization of E13.5 olfactory epithelium fails to detect expression of olfactory neuroepithelial markers in Foxg1-$Cre^{-/-}$ olfactory placodes indicating the failure of olfactory neuronal differentiation in the absence of Foxg1 function.

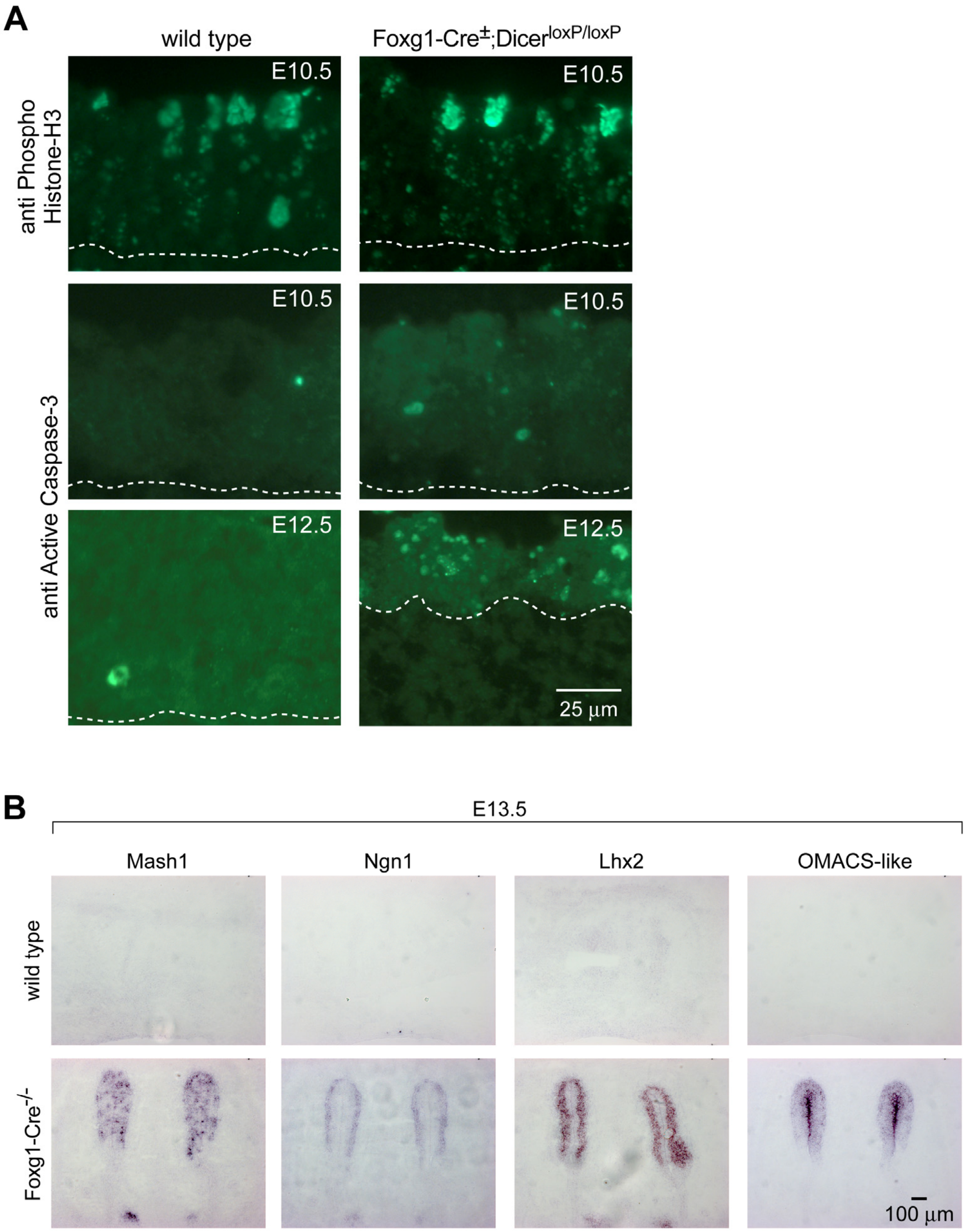
A
wild type
Foxg1-Cre±;DicerloxP/loxP
anti Phospho Histone-H3
anti Active Caspase-3
E10.5
E12.5
25 μm
B
E13.5
Mash1
Ngn1
Lhx2
OMACS-like
wild type
Foxg1-Cre-/-
100 μm

**Supplementary Figure 3. The miR-200 Family is Conserved Between Mouse and Zebrafish at the Genomic, Sequence and Expression Levels**

Comparative genomic analysis of the miR-200 family in mouse and zebrafish indicates extensive conservation with respect to the relative genomic clustering position, the seed region sequences, the size of the family and the arm of the hairpin that generates the mature miRNA.

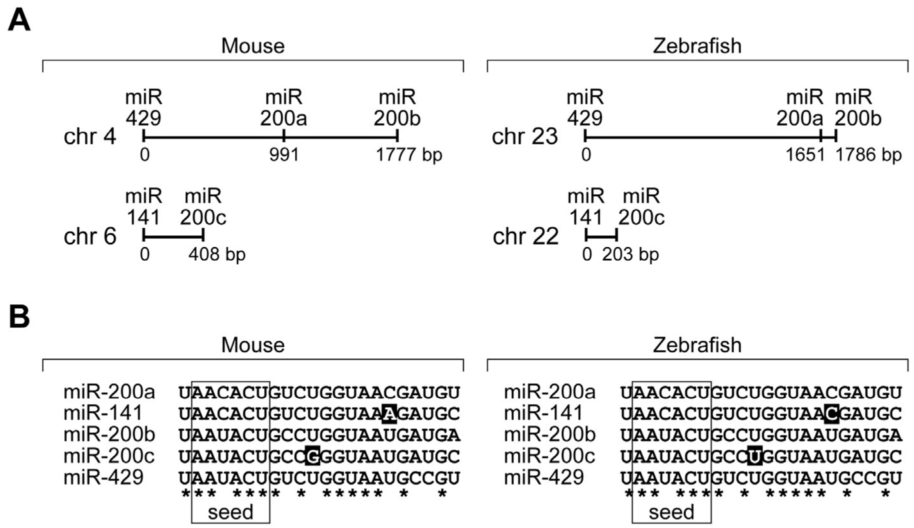

**Supplementary Figure 4. Anti-Sense Morpholino Oligos Specifically and Efficiently Knock Down Expression of Their Cognate Zebrafish miR-200 Targets**

(A) Sequence alignments of synthesized antisense morpholino oligos directed against various miR-200 family members and their corresponding miR-200 family members. miR-141 MO is predicted to specifically hybridize to miR-141 and miR-200a, miR-200b MO is predicted to specifically hybridize to miR-200b and miR-200c and miR-429 MO is predicted to specifically hybridize to miR-420.

(B) One-cell embryos were injected with 4 ng of either miR-141 MO, miR-200b MO or miR-429 MO and systematically assayed for expression of miR-200 family members at 48 hpf indicating that the predictions made in (A) are valid.

(C) Wild type and fish injected with various morpholinos at 48 hpf are morphologically indistinguishable from each other.

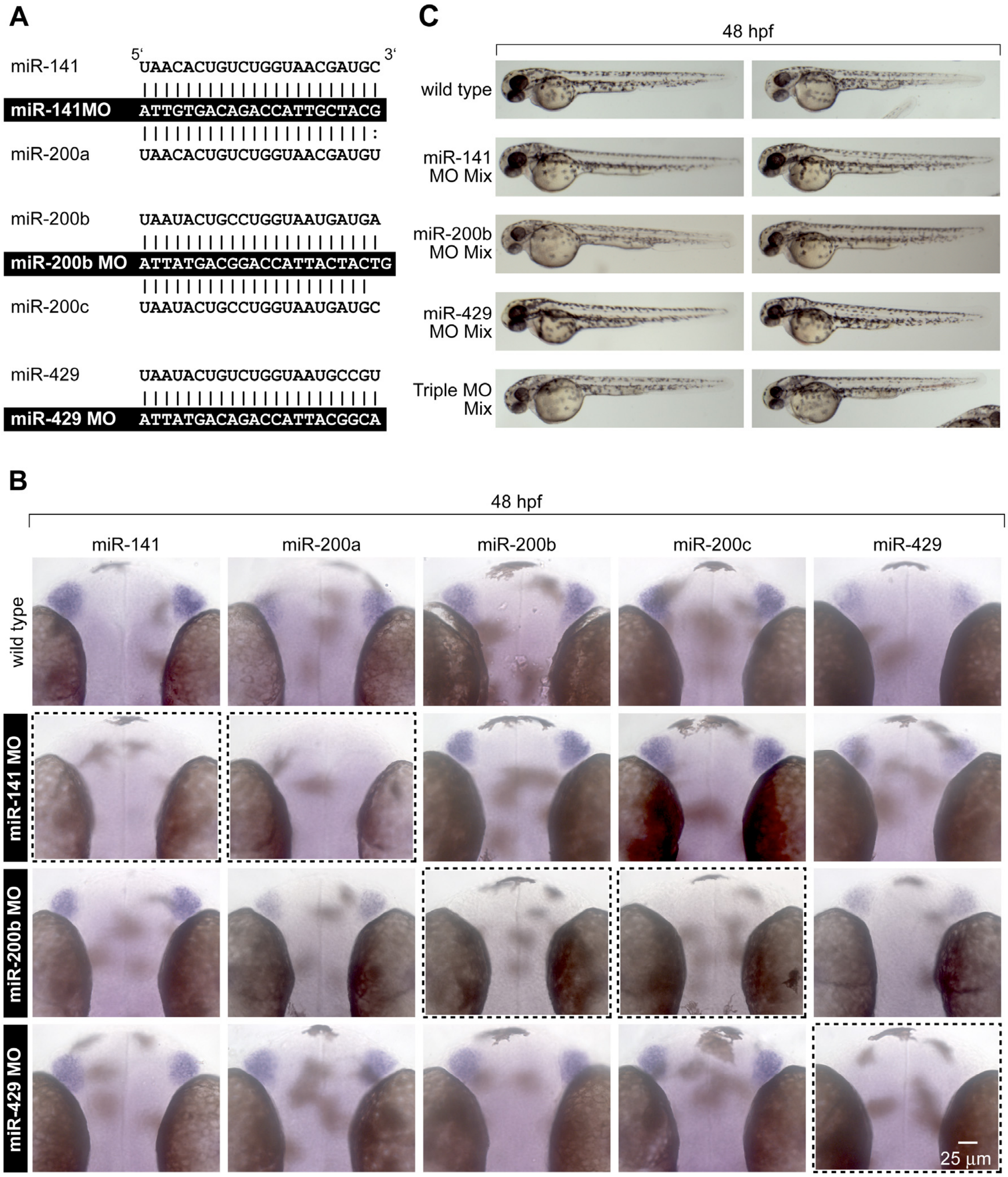
A
miR-141
5' UAACACUGUCUGGUAACGAUGC 3'
miR-141MO
ATTGTGACAGACCATTGCTACG
miR-200a
UAACACUGUCUGGUAACGAUGU
miR-200b
UAAUACUGCCUGGUAAUGAUGA
miR-200b MO
ATTATGACGGACCATTACTACTG
miR-200c
UAAUACUGCCUGGUAAUGAUGC
miR-429
UAAUACUGUCUGGUAAUGCCGU
miR-429 MO
ATTATGACAGACCATTACGGCA
C
48 hpf
wild type
miR-141 MO Mix
miR-200b MO Mix
miR-429 MO Mix
Triple MO Mix
B
48 hpf
miR-141
miR-200a
miR-200b
miR-200c
miR-429
wild type
miR-141 MO
miR-200b MO
miR-429 MO
25 µm
